# Safety and toxicological evaluation of a synthetic vitamin K2, menaquinone-7

**DOI:** 10.3109/15376516.2011.568983

**Published:** 2011-07-25

**Authors:** Kresimir Pucaj, Henrik Rasmussen, Mona Moller, Tom Preston

**Affiliations:** 1 Nucro Technics, Scarborough, Ontario, Canada; 2 Norwegianl Institute of Public Health, Oslo, Norway; 3 Kappa Bioscience AS, Oslo, Norway

**Keywords:** Menaquinone-7, acute oral toxicity, 90-day oral subchronic toxicity study, histopathology

## Abstract

Menaquinone-7 (MK-7) is part of a family of vitamin K that are essential co-factors for the enzyme γ-glutamyl carboxylase, which is involved in the activation of γ-carboxy glutamate (Gla) proteins in the body. Gla proteins are important for normal blood coagulation and normality of bones and arteries. The objective of this study was to examine the potential toxicity of synthetic MK-7 in BomTac:NMRI mice and in Sprague-Dawley rats. In an acute oral toxicity test, mice were administered a single oral dose of 2000 mg/kg body weight (limit dose) and no toxicity was observed during the 14-day observation period. In the subchronic oral toxicity test in rats, animals were administered MK-7 for 90 days by gavage at the following doses: 0 (vehicle control, corn oil), 2.5, 5, and 10 mg/kg body weight/day. All generated data, including clinical observations, ophthalmology, clinical pathology, gross necropsy, and histopathology, revealed no compound-related toxicity in rats. Any statistically significant findings in clinical pathology parameters and/or organ weights noted were considered to be within normal biological variability. Therefore, under the conditions of this experiment, the median lethal dose (LD_50_) of MK-7 after a single oral administration in mice was determined to be greater than the limit dose level of 2000 mg/kg body weight. The no observed adverse effect level (NOAEL) of MK-7, when administered orally to rats for 90 days, was considered to be equal to 10 mg/kg body weight/day, the highest dose tested, based on lack of toxicity during the 90-day study period.

## Introduction

Menaquinone-7 (MK-7) is part of a family of vitamin K, micronutrients necessary for the synthesis of blood coagulation factors and the activation of proteins involved in the building of bones and inhibition of vascular calcification (Cranenburg et al., 2007; Shearer and Newman, 2008).

The vitamin K consists of two main subfamilies: vitamin K1 (phylloquinone) and vitamin K2 or menaquino-nes. All the K vitamins have a similar structure; they share a “quinone” ring and an isoprenoid side chain. The difference lies in the side chain. Menaquinones are classified according to the length of their aliphatic side chain and are designated MK-*n*, where *n* represents the number of isoprenoid residues in that chain (Booth and Suttie, 1998; Shearer and Newman, 2008). Thus, MK-7 contains seven isoprenoid units. The vitamins K are highly lipophilic; MK-7 being more lipophilic than K1 and MK-4, resulting in a much longer half-life (Schurgers and Vermeer, 2002; Schurgers et al., 2007).

Vitamin K plays a critical role as a co-factor for γ-glutamyl carboxylase-catalyzed reactions in the posttranslational carboxylation of glutamic acid to γ-carboxy glutamic acid, and both vitamin K1 and K2 function in this way (Shearer and Newman, 2008). These γ-carboxy glutamate (Gla) residues form calcium-binding sites that are essential for the activity of the proteins in which they are found (Rishavy et al., 2004). Gla-containing proteins are found in bone, and for this reason vitamin K may play an additional role in the homeostasis of bone metabolism and contribute to bone health (Binkley et al., 2002; Knapen et al., 2007; Biigel, 2008; Shea and Booth, 2008), as well as play a role in reducing fracture incidences in postmenopausal women (Cockayne et al., 2006; Iwamoto et al., 2006; Cheung et al., 2008; Iwamoto et al., 2009). In this respect, dietary intake of fermented soybean (natto), which is rich in MK-7, has been associated with reduced bone loss in postmenopausal women (Ikeda et al., 2006). Interestingly, recent data also point to a role of menaquinones and especially the higher menaquinones, such as MK-7, in reducing the incidences of coronary heart disease (CHD) (Geleijnse et al., 2004; Beulens et al., 2009; Gast et al., 2009). Vitamin K1, however, was not shown to have any impact on CHD.

Natto-derived MK-7 is produced by *Bacillus subtilis natto* and consists of all-*trans* MK-7 (Sato et al., 2001), and we were able to produce a synthetic MK-7. Considering the lack of standard toxicological testing on MK-7, the objective of the present study was to determine the safety of MK-7 produced by synthetic means. The potential toxicity of MK-7 was assessed in an acute oral toxicity test in mice and in a subchronic toxicity study in rats following daily oral administration for 90 days (with inclusion of groups allowed to recover for 30 days after the end of dosing).

## Materials and methods

### Test materials

Synthetic all-*trans* MK-7 (molecular weight: 649.0; molecular formula: C_46_H_64_O_2_) was obtained from Kappa Bioscience AS (Oslo, Norway) (produced by Synthetica AS, Oslo, Norway) and is named KB-MK-7 (previously designated as SynMK-7). The MK-7 test article was a yellow powder with 95% to 97% purity (three batches) and was characterized by mass spectrometry (MS), infrared (IR) spectroscopy, high-performance liquid chromatography (HPLC)/ultraviolet (UV) light analysis (270 nm), and nuclear magnetic resonance (NMR). The characteristics of MK-7 are given in Table 1. The product is light-sensitive, and thus, all stock suspensions/solutions and dosing formulations in the acute and subchronic toxicity studies were prepared in the dark either under gold-source fluorescent illumination (wavelength ≥ 520 nm) or in a darkened room with minimal exposure to regular fluorescent lighting. In the subchronic toxicity study, all MK-7 stock solutions and dosing formulations were stored protected from light at 2°C to 8°C. The vehicle control was stored under the same conditions as the MK-7 dosing formulations.

**Table 1:** Technical and chemical description of synthetic menaquinone-7 (MK-7).

Technical and chemical description of synthetic MK-7	
Chemical name(s)	(all-E)-2-(3,7,11,15,19,23,27-Heptamethyl-2,6,10,14,18,22,26-octacosaheptaenyl)-3-methyl-1,4-naphthalenedione
Synonyms	Vitamin MK- 7, Menaquinone-7, Menaquinone 7, Vitamin K2 (35), MK-7
CAS number	2124-57-4
Chemical structure	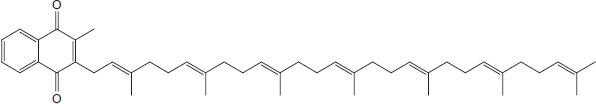
Molecular formula	C_46_H_64_O_2_
Molecular weight	649 g/mol
Appearance	Yellow powder
Odor	Odorless
Melting point	54°C
Boiling point	720.1°C at 760 mm Hg
Flash point	254.9°C
Density	0.961 g/cm^3^
Solubility	Insoluble in water
Purity (HPLC)	≤95% all-*trans* MK-7

Sunflower oil was obtained from Sigma for use as the vehicle in the acute toxicity study. Corn oil was used as the vehicle in the subchronic toxicity study and was obtained from Professional Compounding Centers of America (PCCA, Houston, TX).

### Acute oral toxicity study in female mice

The acute oral toxicity study was conducted in accordance with the Organisation for Economic Cooperation and Development (OECD) Test Guideline 425 for the Testing of Chemicals (The Limit Test). The study protocol was approved by the Norwegian Animal Research Authority and the study was performed at The Norwegian Institute of Public, Department of Laboratory Animal Service Unit, Oslo, Norway, in compliance with the European Convention for the Protection of Vertebrate Animals Used for Experimental and Other Scientific Purposes (ETS No. 123, 1986). The study was performed in the spirit of Good Laboratory Practice (GLP).

#### Preparation of dosing formulation

MK-7 was dissolved in sunflower oil to a nominal working concentration of 200 mg/mL. The MK-7 dosing suspension was prepared fresh and delivered to the animal facility on the day of use. The test substance was heated in a water bath of ∼37°C and was transparent and clear without particulates upon aspiration and dosing. The MK-7 dosing suspension was protected from light until aspiration into the dosing syringe. The results for calculated dose concentrations of vitamin MK-7 in sunflower oil were within ±10% of the target dose, quantified using a reversed phase (HPLC-UV) assay method.

#### Animals and treatment

Nulliparous and non-pregnant female BomTac:NMRI mice were obtained from Taconic (Ry, Denmark), and were 8 weeks at the start of the study. Five animals out of the available seven animals were randomly selected at study start with weight as the limiting factor. The animals were specific pathogen-free according to the Federation of European Laboratory Animal Science Associations guidelines and were clinically examined upon arrival and identified by ear marking. The animals were allowed an acclimatization period of 6 days after delivery and before they entered the study. They were housed in standard type III macrolon cages in a Scantainer cabinet under controlled temperature and light conditions (22±3°C, 40-70% relative humidity, 12-h light phase with daylight) with *ad libitum* access to SDS RM1 diet (Special Diet Services, UK) and municipal water (food was temporarily withdrawn for 1-2 h prior to oral gavage on Day 0). Nestpak aspen tree bedding (Datesand Ltd., UK) and cage enrichment (plastic igloos) were used. The animals were housed in groups of 3 or 4 per cage during acclimatization and until the day of dosing. The animals were housed as single animals during the first 48 h after dosing. Following the 48-h observation period for animals 2 to 5, all animals were returned to their original groups and the animals were housed in groups of three or four animals for the remainder of the 14-day observation period.

The animals were monitored for the first 30 min after dosing and twice daily on the day of dosing. Observations were done once daily thereafter for the remainder of the 14-day observation period. In addition to mortality or killing of animals for humane welfare reasons, observations included clinical signs indicative of changes from normality with respect to skin, fur, eyes, mucous membranes, and respiratory, circulatory, autonomic, and central nervous systems. Animals were also observed for changes in motor activity and behavior pattern. The mice were sacrificed by cervical dislocation at the end of the observation period.

MK-7 was administered by oral gavage, using a stainless steel gavage needle. The animals were dosed with 10 mL/kg body weight of the supplied suspension, corresponding to a dose of 2000mg/kg body weight (based on a target concentration of 200 mg/mL). Animal number 1 was dosed first and animals 2 to 5 were dosed 48 h later. The day of dosing was designated as Day 0 and animals were weighed at Days 0, 7, and 14 (termination).

#### Observations

The animals were monitored twice daily on the day of dosing and once daily thereafter for the remainder of the 14-day observation period. Observations included changes in skin, fur, eyes, mucous membranes, and respiratory, circulatory, autonomic, and central nervous systems. Animals were also observed for changes in motor activity and behavior pattern. The mice were sacrificed by cervical dislocation at the end of the observation period.

### The 90-day subchronic toxicity study in rats

The 90-day toxicity study was performed in accordance with the United States Food and Drug Administration (U.S. FDA) regulations on GLP for Nonclinical Laboratory Studies (Title 21, Part 58 of the Code of Federal Regulations) and with the OECD Principles of GLP. The study design was based on the principles of the current test guidelines for repeated-dose toxicity studies as issued by the U.S. FDA, OECD (Test Guideline 408), Health Canada (Therapeutics Product Directorate), and the International Conference on Harmonisation (ICH) [Guideline M3(R2)]. The study was conducted at Nucro-Technics, Toronto, ON, Canada. The GLP compliance of this 90-day study as attested by the signatures of the Study Director and the Institutional Quality Assurance Unit indicated that the methods, results, and data contained in the 90-day study report accurately reflect the procedures followed and raw data collected during the study.

#### Preparation of dosing formulations

For the 90-day subchronic toxicity study, the MK-7 test article was produced according to Good Manufacturing Practice (GMP). Dosing formulations, consisting of a stock solution of 2.0 mg/mL MK-7 in corn oil and stock solution dilutions of 1.0 and 0.5 mg/mL MK-7 in corn oil, were prepared every 3 to 4 days and aliquoted daily prior to use. The purity of the test article was taken into account during preparation. The mean dosing concentrations of dosing formulations prepared on Days 1, 30, 59, and 90 were analytically determined by LC/MS/MS to be within 92.5 ± 4.6%, 92.5 ± 4.6%, and 95.9 ± 8.7% of the nominal concentrations for the 0.5, 1, and 2 mg/mL dose formulations, respectively. During dose administration, all MK-7 dosing formulations were continually mixed on a stir plate and protected from light until aspiration into the dosing syringe. The corn oil vehicle control was aliquoted and handled under the same conditions.

#### Animals and treatment

Animals used in this study were acclimatized for 3 weeks and treated and cared for in accordance with the guidelines recommended by the Canadian Council on Animal Care (CCAC) and the Association for Assessment and Accreditation of Laboratory Animal Care International (AAALAC), and the experimental protocol for treating the animals was approved by the Institutional Animal Care Committee of Nucro-Technics under the Protocol number: 220117.AUP. A total of 70 male and 71 female Sprague-Dawley rats (*Rattus norvegicus*), aged 4-5 weeks, were obtained from Charles River Canada Inc., Montreal, PQ. Each animal was identified with a unique tail tattoo. At the start of dosing, males weighed between 259 and 329 g and females weighed between 190 and 227 g. The rats were housed individually in Nalgene” rat cages with stainless steel cage covers under controlled conditions: temperature of 18°C to 26°C, relative humidity of 30-70%, a minimum of 10 air changes per hour, and a 12-h light and 12-h dark cycle. The animals were provided Teklad Certified Rodent Diet (#8728C) and municipal water (using water bottles) *ad libitum*. All contaminants in the feed and water were confirmed to be within acceptable ranges. On arrival, all rats were subjected to a general physical examination by a qualified technician to ensure that they were free of disease. Animals were acclimated over a 21-day period, during which time they were observed for clinical signs of disease. In addition, a health screen was performed on five male and five female rats that were randomly selected from the rat colony. At the end of the acclimatization period, all remaining rats were found to be healthy and 50 rats of each sex were randomized into four groups using randomization statistical software. Each group consisted of 10 rats of each sex with an additional five rats/sex allotted to the control and high-dose recovery groups.

Animals were administered MK-7 at a dose of 0 (vehicle corn oil only), 2.5 (low-dose), 5.0 (mid-dose), or 10.0 mg/kg body weight/day (high-dose) by oral gavage for 90 consecutive days. The dosing volume administered to each animal was 5 mL/kg body weight, adjusted based on the animal's most recent body weight. The dose levels were chosen based on the anticipated daily dose in humans and on the results from the 28-day dose range finding study in which no compound-related adverse effects were observed at doses of up to 10 mg/kg body weight/day.

#### Clinical observations, body weights, and food consumption

Animals were monitored closely after dosing, and were observed for clinical signs and mortality twice daily throughout the study. Animals were observed for reaction to treatment such as changes in skin, fur, eyes, and mucous membranes. Respiratory, circulatory, autonomic, central nervous system, somatomotor activity, and behavior patterns were also monitored along with any other signs of ill-health. Detailed clinical signs were recorded once a week, with examination of general appearance, respiration, abnormalities of behavior and movement, external organs, skin, and any lesions. A functional observation battery (FOB) assessment for motor activity, grip strength, and sensory activity to visual, audio, and proprioceptive stimuli was conducted on all animals during the last week of dosing and on recovery animals during the last week of the study.

Body weights were recorded during the acclimatization period (Days -19, -12, and -5), on the first day of administration (Day 1) prior to dosing, and then weekly thereafter. End of treatment body weights (unfasted) were recorded on Day 90. Terminal body weights of main study rats were recorded prior to necropsy on Day 91 or 92 following an overnight fast. Body weights of recovery animals were recorded on Days 1, 8, 15, 22, 30, and prior to necropsy (fasted) on Day 31 of the recovery period (Day 121 of the study). Body weight changes (unfasted) were calculated. Food consumption was recorded during the acclimatization period and weekly until scheduled necropsy.

Funduscopic (indirect ophthalmoscopy) and biomicroscopic (slit lamp) examinations were performed on all animals ∼2 weeks prior to the start of treatment and on all main study and recovery rats on Day 87. Ophthalmoscopy was not conducted at the end of the recovery period, as there was no indication of ophthalmic toxicity in rats examined at the end of the treatment period.

#### Clinical pathology

Blood samples were collected on Day 44 from five male and five female rats (fasted) by orbital sinus or jugular bleeds and limited clinical pathology (hematology and clinical chemistry) performed. Blood samples were also collected on the day of necropsy from all main study and recovery animals following an overnight fast. Blood was obtained from the abdominal aorta following anesthesia induced by isoflurane and routine clinical pathology investigations performed. Hematology was performed on EDTA-treated samples and parameters measured included red blood cell (RBC) count, hematocrit, hemoglobin, white blood cell (WBC) count, WBC differential, mean corpuscular hemoglobin (MCH), MCH concentration (MCHC), mean corpuscular volume (MCV), mean platelet volume, cell morphology, platelet count, and reticulocyte count. Coagulation was determined from citrate-treated samples and parameters measured included activated partial thromboplastin time (APTT) and prothrombin time (PT). Clinical chemistry was performed on serum and included total protein, albumin, globulin (calculated), albumin/globulin (A/G) ratio, alanine aminotransferase (ALT), alkaline phosphatase (ALP), aspartate aminotransferase (AST), γ-glutamyl transferase (GGT), lactate dehydrogenase (LDH), creatine kinase (CK), total bilirubin, blood urea nitrogen (BUN), creatinine, fasting glucose, total cholesterol, triglycerides, calcium, chloride, inorganic phosphorus, potassium, and sodium. Hematology and clinical chemistry data were compared with the laboratory's baseline historical data for Sprague-Dawley rats.

Urine was collected overnight (approximately a 12-h period) by placing rats in metabolic cages during the last weeks of dosing and recovery. Animals were fasted during urine collection. The urine samples were analyzed for color, appearance, volume, pH-specific gravity, bilirubin, urobilinogen, blood, glucose, ketones, leukocytes, nitrite, and sediment microscopy.

#### Pathology

On Day 91 (males) or 92 (females), following an overnight fast (∼ 12 h), all surviving main study animals were anesthetized by exposure to isoflurane, exsanguinated, and subjected to necropsy. Recovery animals were treated similarly on Day 31 of the recovery period. Necropsies consisted of an external examination, including reference to all clinically recorded lesions, and a detailed internal examination. Organs that were collected and weighed included the adrenals, ovaries, brain, pituitary gland, epididymis, prostate, heart, spleen, kidneys, thymus, liver, testes, lungs, and uterus (horns, cervix, and body). Paired organs were weighed together, and absolute and relative weights (relative to terminal body weight and brain weight) were calculated. The following organs and tissues were also collected: aorta (thoracic), cecum, colon, duodenum, esophagus, ileum, jejunum, liver (sample of central and left lobes), lymph nodes (mandibular and mesenteric), mammary glands (inguinal), salivary glands (submandibular), seminal vesicles, sciatic nerve, skeletal muscle (quadriceps), skin (inguinal) and subcutis, spinal cord, sternum and marrow, stomach, thyroid/parathyroids, and urinary bladder. Organs and tissues were fixed in 10% neutral buffered formalin, except for the eyes, optic nerves, and testes, which were fixed in alcoholic formalin, paraffin-embedded, sectioned, and stained with hematoxylin and eosin. Full histo-pathological examination was performed on tissues from control and high-dose animals, as well as on tissues with abnormal findings from all dose groups.

### Statistical analysis

For the 90-day toxicity study homogeneous data were analyzed using the analysis of variance (at *P* = 0.05), and the significance of intergroup differences were analyzed using Duncan's or other appropriate test. Heterogeneous data were analyzed using the Kruskal-Wallis test and the significance of intergroup differences between the control and treated groups were assessed using Dunn's or other appropriate test. T-test was conducted on the recovery rats.

## Results

### Acute oral toxicity study in mice

At the limit dose level of 2000 mg/kg body weight, MK-7 did not induce any signs of toxicity in any of the treated mice following dosing and during the 14-day observation period. In addition, the body weight gain of treated mice was not adversely affected. Two non-treated animals were included as a reference. Based on these results and under the conditions of this study, the median lethal dose (LD_50_) of MK-7 was determined to be greater than the limit dose level of 2000 mg/kg body weight in BomTac:NMRI mice.

### The 90-day subchronic toxicity study in rats

#### Clinical observations, body weights, and food consumption

All animals from the main study and recovery groups survived the course of the study with no clinical signs of systemic toxicity or abnormalities in neurological function. Alopecia was observed in one low-dose and one high-dose female, the latter of which also displayed reduced body weight gain. Both animals were otherwise healthy with no other abnormalities noted in all other parameters assessed. One male rat of the low-dose group presented with clinical signs (dyspnea, piloerection, and lameness), reduced food consumption, and loss in body weight, which were attributed to a procedural injury, consisting of deposition of the test article in the periesophageal tissue. Consequently, an abscess had formed in the left upper thorax region (confirmed at necropsy and histopathology examination), which was surgically drained on Day 70 following an 8-day suspension of test article administration. Body weight gain was subsequently observed in this animal. Additionally, inclusion of this animal in the group food consumption, body weight, and body weight gain results did not result in statistically significant differences compared with the control group.

At the end of the dosing period, ophthalmological examinations revealed corneal-subepithelial crystalline opacity in all groups, including the control group, with similar frequency and severity among groups. Cataracts were also noted in groups administered MK-7; however, a dose-dependent response was not observed. All ophthalmological observations were within historical ranges for the strain and age of rats.

**Figure 1:**
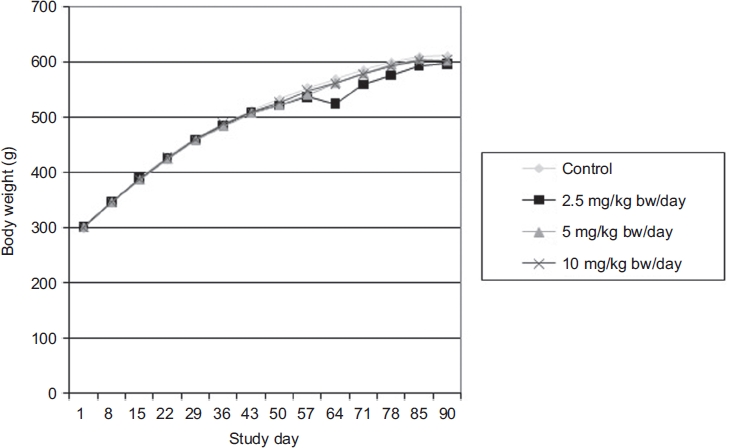
Body weight of Sprague-Dawley male rats as a function of time following administration of menaquinone-7 (MK-7) in the 90-day subchronic toxicity study. Each value represents the mean of 10 animals.

**Figure 2:**
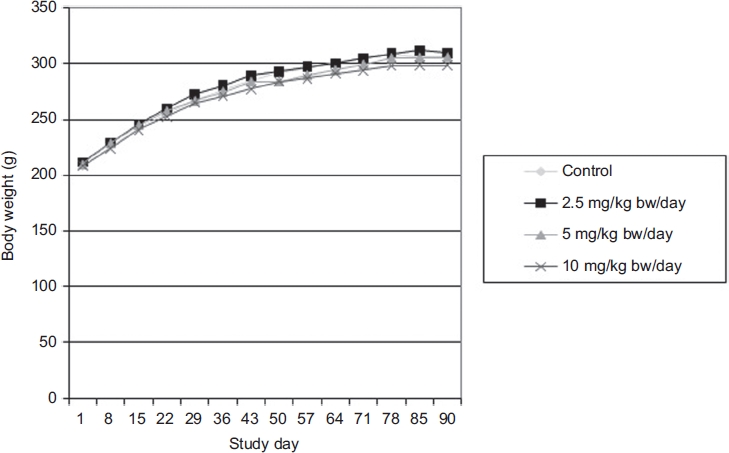
Body weight of Sprague-Dawley female rats as a function of time following administration of menaquinone-7 (MK-7) in the 90-day subchronic toxicity study. Each value represents the mean of 10 animals.

All groups displayed satisfactory body weight gains and no significant differences in body weights or body weight gains were observed between the control and MK-7 test groups (Figures 1 and 2). Food consumption was also not significantly different between the control and MK-7 test groups (data not shown). Water consumption, monitored daily by visually observing water levels in bottles, did not appear to differ between groups (actual water consumption was not measured).

#### Clinical pathology

Significant differences in hematology results included an increase in MCV in mid-dose females at mid-study (Day 44) and an increase in hematocrit in high-dose males of the recovery group compared with control rats (data not shown). These changes were neither dose- nor time-dependent, were not observed at any other time point or in the opposite sex, and were slight in magnitude. No other significant differences in hematology were observed between the control and MK-7 test groups. At mid-study, several parameters in various groups, including control animals, were slightly above the upper limit of the normal ranges, with no dose-dependency observed. At the end of the dosing and recovery periods, the results of all hematology parameters examined were within normal baseline historical ranges in all groups (data not shown).

Coagulation time group summaries are presented in Table 2. A significant increase in APTT was observed in female rats of the mid- and high-dose groups at the end of the dosing period compared with the control group. The change in APTT was resultant of two rats in the mid-dose group and one rat in the high-dose group with elevated APTT times (i.e. the two female rats from the mid-dose group had APTT values of 29.2 sec and 29.6 sec and the high-dose female had an APTT value of 38.8 sec). When these rats were removed from the calculations, the APTT was comparable among all groups, with no statistically significant differences. A similar change was not observed in male rats and the mean PT and APTT were within the normal physiological limits at the end of the dosing and recovery periods in all groups.

**Table 2:** Coagulation parameters in male and female rats following oral administration of menaquinone-7 (MK-7) in the 90-day subchronic toxicity study.

		Males^a^				Females^a^			
			
Parameter	Study day	Control	2.5 mg/kg bw/d	5 mg/kg bw/d	10 mg/kg bw/d	Control	2.5 mg/kg bw/d	5 mg/kg bw/d	10 mg/kg bw/d
Prothrombin time (sec)	Day 90	19.2 ± 1.4	19.2 ± 0.6	19.2 ± 1.2	19.0 ± 0.8	17.9 ± 0.7	18.5 ± 0.9	17.4 ± 1.0	17.2 ± 0.9
	Day 121 (recovery)	20.9 ± 1.5	n/a	n/a	20.0 ± 0.9	19.2 ± 1.3	n/a	n/a	18.9 ± 0.6
APTT (sec)	Day 90	19.4 ± 3.2	19.0 ± 3.2	19.0 ± 2.3	22.5 ± 5.1	19.3 ± 1.2	20.7 ± 1.6	22.6 ± 3.6*	23.6 ± 6.1*
	Day 121 (recovery)	17.4 ± 3.1	n/a	n/a	16.3 ± 1.8	17.3 ± 2.1	n/a	n/a	17.1 ± 0.3

a Only the control and high-dose (10 mg/kg bw/d) groups were included in the 30-day recovery period of the study. All groups consisted of 10 animals, with the exception of the recovery groups (5 animals /group).

* Statistically significant different from the control group (*P* < 0.05).

Clinical chemistry results revealed a few incidental significant differences between control and MK-7 test groups at mid-study and at the end of the dosing period as shown in Table 3. These changes were neither dose- nor time-dependent, were observed at only one time point, and were, for the most part, observed in one sex only. These changes were typically within the normal ranges, with a few significantly different results that were very slightly above the upper limit of the normal ranges in female rats at the mid-study point only. Some other values, including those of the control group, were also observed to be at the high or low end of the physiological range, but without dose- or time-dependency. Apart from a significant, yet slight, increase in serum calcium concentration in high-dose males, no significant differences were observed in clinical chemistry results compared with controls at the end of the recovery period in either males or females (data not shown). Urinalysis results were also unremarkable, with no differences observed between control and MK-7 test groups (data not shown).

**Table 3:** Clinical chemistry parameters in male and female rats following oral administration of menaquinone-7 (MK-7) in the 90-day subchronic toxicity study.

		Males^a^				Females^a^			
			
Parameter	Study day	Control	2.5 mg/kg bw/d	5 mg/kg bw/d	10 mg/kg bw/d	Control	2.5 mg/kg bw/d	5 mg/kg bw/d	10 mg/kg bw/d
A/G	Day 44	1.3±0.1	1.6±0.1*	1.5 ±0.2*	1.3±0.1	1.5±0.2	1.7±0.1	1.9 ±0.3*	1.5±0.1
	Day 90	1.2±0.1	1.2±0.1	1.2±0.1	1.2±0.1	1.4±0.1	1.4±0.1	1.4±0.1	1.4 ±0.2
ALB (g/L)	Day 44	36±4	42±3	42±6	36±4	40±6	46±3	50 ±5*	41±4
	Day 90	32±2	32±2	33±2	31±1	37±2	38±4	38±3	38±6
GLOB (g/L)	Day 44	27±1	27±2	28±1	27±1	27±1	27±1	27±1	28±1
	Day 90	28±1	28±1	27±1	26 ±2	27±2	27±2	27±1	27±1
ALP (U/L)	Day 44	199 ±59	180±44	183 ±44	164 ±47	87 ±13	136±48	140±31	119±25
	Day 90	117±33	111±23	111±33	97 ±28	62 ±17	72 ±28	63 ±18	76 ±17
Bil (T) (μmol/L)	Day 44	3.1±0.8	5.1 ±2.2	5.3 ±2.5	4.1±2.1	2.7±0.9	3.4±1.1	5.4±2.5	3.6±1.0
	Day 90	2.6±0.6	2.9±0.5	3.1±1.2	2.9±0.6	4.9±1.2	4.9±1.5	4.6±0.8	4.5 ±1.3
BUN (mmol/L)	Day 44	3.9±0.8	4.1 ±1.3	3.9±0.8	3.7±0.6	3.7±0.7	3.9±0.7	4.6±0.9	4.1 ±0.3
	Day 90	4.3±0.9	4.6 ±0.7	4.4 ±0.7	4.3 ±0.6	4.9±0.7	5.1±0.7	4.9±0.4	5.0 ±1.2
Ca (mmol/L)	Day 44	2.64 ±0.08	2.62 ±0.08	2.55±0.12	2.54 ±0.09	2.69 ±0.09	2.72 ±0.06	2.86±0.17	2.68±0.16
	Day 90	2.47 ±0.08	2.53 ±0.08	2.54 ±0.08	2.50 ±0.08	2.51±0.06	2.53 ±0.05	2.57±0.06	2.56±0.12
Cl (mmol/L)	Day 44	102 ±2	105±2*	107 ±2*	104 ±1	104±3	109 ±2*	111±3*	105±2
	Day 90	103 ±2	103 ±2	103 ±1	104±2	104 ±1	105±2	104 ±2	105±3
Creatinine (μmol/L)	Day 44	49 ±11	53±11	48±15	41±6	41±7	44±9	52 ±17	39 ±3
	Day 90	44±4	42 ±3	41±4	42±3	44±2	42±3	41±2	43 ±2
Glucose (mmol/L)	Day 44	5.3 ±0.7	7.6±2.3*	6.4 ±0.8	5.2 ±0.7	5.5±0.8	5.9 ±0.4	5.9 ±0.8	5.6±0.6
	Day 90	10.2±2.1	11.4±2.6	11.6±2.2	10.3±2.2	8.8±1.9	10.5 ±1.4*	8.8 ±1.4	10.4 ±1.5
LDH (U/L)	Day 44	2487 ±1487	2056 ±721	2941 ±1066	4904 ±3338	1002 ±443	2349 ±959	1462 ±419	2026 ±996
	Day 90	4837 ±2285	3897±2604	4783±2201	3856 ±2730	5496±2219	2805 ±1709*	3659 ±1802	3168±1735
P (mmol/L)	Day 44	2.81±0.46	3.02 ±0.44	2.95 ±0.69	2.48 ±0.30	2.10±0.26	2.25 ±0.35	2.64 ±0.74	2.08 ±0.22
	Day 90	2.03 ±0.2	2.04±0.19	2.15±0.24	2.06±0.16	1.78±0.2	1.88 ±0.24	1.87±0.16	1.86 ±0.23
K (mmol/L)	Day 44	6.5±0.9	6.9 ±0.4	7.1±1.2	6.0 ±0.4	5.6±0.9	6.1±0.8	7.3 ±1.4	6.0±0.7
	Day 90	5.2 ±0.2	5.0 ±0.4	5.1±0.3	4.9 ±0.3	4.6±0.3	4.5 ±0.4	4.5 ±0.2	4.4 ±0.3
Protein (T) (g/L)	Day 44	64±4	70±4	70±7	63±5	67±6	73 ±3	80 ±8*	69±5
	Day 90	60±2	60±2	59±3	57 ±2*	64±2	65 ±4	65±3	65±7
AST (U/L)	Day 44	84 ±16	107±21	102±10	115±23	69 ±11	94 ±19	87 ±14	83 ±13
	Day 90	104±25	109 ±53	101±19	105±29	110±30	83 ±18*	89±21	90 ±12
ALT (U/L)	Day 44	35±4	43±3	39±9	42±8	37±4	38±3	40 ±12	41±9
	Day 90	40±5	54 ±42	42±6	44±9	36±5	35±11	35±3	40±7
Na (mmol/L)	Day 44	145 ±5	149±4	153 ±5	146±3	145 ±4	151±5	158±4*	146±3
	Day 90	141±2	141±1	142 ±1	142 ±1	141±2	142 ±2	142 ±1	142 ±2
Triglycerides (mmol/L)	Day 44	0.69 ±0.28	0.93 ±0.38	0.67±0.21	0.66 ±0.32	0.45 ±0.09	0.49 ±0.04	0.57 ±0.24	0.45 ±0.06
	Day 90	0.72 ±0.31	0.83 ±0.39	0.65±0.16	0.53±0.11	0.50±0.09	0.59±0.12	0.53 ±0.09	0.81 ±0.42*
CK (U/L)	Day 44	309±150	534 ±356	401 ±93	531 ±252	104±26	301±97	222 ±163	182 ±79
	Day 90	454 ±185	338 ±221	416±166	349 ±182	481 ±162	285 ±168*	342 ±152	247 ±112*
Cholesterol (mmol/L)	Day 44	2.73 ±0.27	2.79 ±0.45	3.02 ±0.52	2.31±0.56	2.32 ±0.60	2.81±0.47	3.47 ±0.86	2.93 ±0.83
	Day 90	2.43 ±0.38	2.60 ±0.66	2.54 ±0.52	2.11±0.46	2.48 ±0.65	2.43 ±0.67	2.64 ±0.42	2.47 ±0.86
GGT (U/L)	Day 44	<5±0	<5±0	<5±0	<5±0	<5±0	<5±0	<5±0	<5±0
	Day 90	<5±0	<5±0	<5±0	<5±0	<5±0	<5±0	<5±0	<5±0

ALB = albumin; ALP = alkaline phosphatase; ALT = alanine aminostranferase; AST = aspartate aminotransferase; Bil = bilirubin; BUN = blood urea nitrogen; Ca = calcium; Cl = chloride; CK = creatine kinase; d = day; GGT = gamma-glutamyl transpeptidase; LDH = lactate dehydrogenase; K = potassium; P = phosphorus; LDH = lactate dehydrogenase; K = potassium; P = phosphorus.

* Statistically significant difference from control (*P* < 0.05).

a All groups consisted of 10 animals.

#### Pathology

Organ weights and organ to body and brain weight ratio summaries are presented in Table 4. No significant differences in absolute or relative organ weights were observed between male control and MK-7 test groups at the end of the dosing and recovery periods. In female rats administered MK-7, the following significant differences were observed compared with control females at the end of the dosing period: a decrease in absolute and relative (to body and brain weight) ovary weight in the mid-dose group and an increase in lung weight relative to brain weight in the high-dose group. At the end of the recovery period, the following significant differences were observed in the high-dose female group: a decrease in absolute and relative (to body and brain weight) thymus weight, a decrease in relative (to brain weight) spleen weight, and an increase in brain weight relative to body weight. No dose-dependent relationship was observed in the organ weight changes and all changes remained within or at the limits of the normal historical ranges.

**Table 4:** Organ weights of male and female rats following oral administration of menaquinone-7 (MK-7) in the 90-day subchronic toxicity study.

		Males^a^				Females^a^			
			
Organ	Study day	Control	2.5 mg/kg bw/d	5 mg/kg bw/d	10 mg/kg bw/d	Control	2.5 mg/kg bw/d	5 mg/kg bw/d	10 mg/kg bw/d
Absolute organ weight (g)									
Spleen	Day 90	0.877 ± 0.479	0.832 ± 0.140	0.836 ± 0.146	0.884 ± 0.181	0.548 ± 0.069	0.546 ± 0.095	0.553 ± 0.091	0.575 ± 0.102
	Day 120 (recovery)	1.008 ± 0.114	n/a	n/a	0.971 ± 0.114	0.629 ± 0.046	n/a	n/a	0.555 ± 0.058
Liver	Day 90	16.050 ± 2.029	16.540 ± 2.03	15.68 ± 2.442	15.520 ± 1.844	7.880 ± 1.081	8.290 ± 0.471	8.00 ± 1.192	7.840 ± 0.517
	Day 120 (recovery)	16.28 ± 1.100	n/a	n/a	16.440 ± 2.648	8.830 ± 1.226	n/a	n/a	7.960 ± 0.810
Adrenal glands	Day 90	0.101 ± 0.041	0.089 ± 0.022	0.090 ± 0.024	0.082 ± 0.016	0.079 ± 0.012	0.083 ± 0.014	0.084 ± 0.020	0.085 ± 0.011
	Day 120 (recovery)	0.081 ± 0.022	n/a	n/a	0.072 ± 0.011	0.089 ± 0.016	n/a	n/a	0.081 ± 0.009
Kidneys	Day 90	3.750 ± 0.469	3.83 ± 0.382	3.680 ± 0.431	3.770 ± 0.406	2.070 ± 0.20	2.080 ± 0.153	2.040 ± 0.242	2.120 ± 0.150
	Day 120 (recovery)	3.860 ± 0.503	n/a	n/a	3.810 ± 0.510	2.080 ± 0.099	n/a	n/a	2.020 ± 0.170
Lungs and trachea	Day 90	2.160 ± 0.239	2.110 ± 0.273	2.190 ± 0.415	2.220 ± 0.266	1.39 ± 0.11	1.36 ± 0.149	1.440 ± 0.121	1.510 ± 0.086
	Day 120 (recovery)	2.030 ± 0.269	n/a	n/a	2.080 ± 0.316	1.430 ± 0.164	n/a	n/a	1.400 ± 0.229
Heart	Day 90	1.646 ± 0.232	1.544 ± 0.099	1.536 ± 0.228	1.52 ± 0.117	0.940 ± 0.069	0.949 ± 0.092	0.920 ± 0.090	0.942 ± 0.070
	Day 120 (recovery)	1.594 ± 0.213	n/a	n/a	1.505 ± 0.172	0.972 ± 0.095	n/a	n/a	0.945 ± 0.089
Thymus	Day 90	0.485 ± 0.112	0.441 ± 0.109	0.463 ± 0.057	0.469 ± 0.094	0.289 ± 0.061	0.278 ± 0.056	0.253 ± 0.044	0.296 ± 0.050
	Day 120 (recovery)	0.476 ± 0.081	n/a	n/a	0.445 ± 0.090	0.385 ± 0.028	n/a	n/a	0.295 ± 0.041**
Brain	Day 90	2.230 ± 0.069	2.210 ± 0.135	2.140 ± 0.127	2.160 ± 0.075	1.98 ± 0.096	1.96 ± 0.094	1.95 ± 0.091	1.920 ± 0.105
	Day 120 (recovery)	2.200 ± 0.088	n/a	n/a	2.160 ± 0.066	1.960 ± 0.04	n/a	n/a	2.000 ± 0.095
Pituitary	Day 90	0.015 ± 0.002	0.013 ± 0.002	0.012 ± 0.002	0.013 ± 0.002	0.015 ± 0.002	0.014 ± 0.002	0.016 ± 0.003	0.014 ± 0.003
	Day 120 (recovery)	0.012 ± 0.003	n/a	n/a	0.014 ± 0.001	0.018 ± 0.003	n/a	n/a	0.017 ± 0.002
Epididymis	Day 90	1.670 ± 0.274	1.760 ± 0.229	1.710 ± 0.179	1.810 ± 0.193	n/a	n/a	n/a	n/a
	Day 120 (recovery)	1.830 ± 0.374	n/a	n/a	1.900 ± 0.656	n/a	n/a	n/a	n/a
Testes	Day 90	4.02 ± 0.296	3.950 ± 0.335	3.740 ± 0.335	3.970 ± 0.429	n/a	n/a	n/a	n/a
	Day 120 (recovery)	3.770 ± 0.269	n/a	n/a	3.810 ± 0.450	n/a	n/a	n/a	n/a
Prostate	Day 90	1.7 ± 0.355	1.42 ± 0.345	1.510 ± 0.347	1.29 ± 0.292	n/a	n/a	n/a	n/a
	Day 120 (recovery)	1.910 ± 0.365	n/a	n/a	1.660 ± 0.394	n/a	n/a	n/a	n/a
Ovaries	Day 90	n/a	n/a	n/a	n/a	0.194 ± 0.034	0.172 ± 0.024	0.142 ± 0.40	0.159 ± 0.049*
	Day 120 (recovery)	n/a	n/a	n/a	n/a	0.225 ± 0.047	n/a	n/a	0.181 ± 0.040
Uterus	Day 90	n/a	n/a	n/a	n/a	0.732 ± 0.203	0.667 ± 0.147	0.796 ± 0.181	0.790 ± 0.137
	Day 120 (recovery)	n/a	n/a	n/a	n/a	1.181 ± 0.646	n/a	n/a	0.898 ± 0.108
Relative organ weight (% bw)									
Spleen	Day 90	0.147 ± 0.017	0.145 ± 0.026	0.143 ± 0.016	0.151 ± 0.021	0.194 ± 0.026	0.185 ± 0.030	0.191 ± 0.031	0.202 ± 0.041
	Day 120 (recovery)	0.166 ± 0.014	n/a	n/a	0.161 ± 0.023	0.195 ± 0.006	n/a	n/a	0.194 ± 0.013
Liver	Day 90	2.690 ± 0.166	2.869 ± 0.250	2.687 ± 0.211	2.658 ± 0.139	2.780 ± 0.334	2.815 ± 0.203	2.746 ± 0.309	2.751 ± 0.271
	Day 120 (recovery)	2.684 ± 0.165	n/a	n/a	2.704 ± 0.318	2.730 ± 0.197	n/a	n/a	2.773 ± 0.145
Adrenal glands	Day 90	0.017 ± 0.008	0.016 ± 0.004	0.015 ± 0.004	0.014 ± 0.003	0.028 ± 0.005	0.028 ± 0.006	0.029 ± 0.007	0.030 ± 0.005
	Day 120 (recovery)	0.013 ± 0.003	n/a	n/a	0.012 ± 0.002	0.027 ± 0.005	n/a	n/a	0.028 ± 0.003
Kidneys	Day 90	0.630 ± 0.055	0.667 ± 0.073	0.634 ± 0.059	0.646 ± 0.038	0.729 ± 0.034	0.705 ± 0.062	0.700 ± 0.046	0.743 ± 0.055
	Day 120 (recovery)	0.634 ± 0.054	n/a	n/a	0.629 ± 0.076	0.647 ± 0.065	n/a	n/a	0.705 ± 0.051
Lungs and trachea	Day 90	0.363 ± 0.038	0.368 ± 0.049	0.382 ± 0.101	0.382 ± 0.42	0.491 ± 0.048	0.460 ± 0.038	0.497 ± 0.027	0.529 ± 0.030
	Day 120 (recovery)	0.334 ± 0.034	n/a	n/a	0.344 ± 0.049	0.446 ± 0.064	n/a	n/a	0.485 ± 0.043
Heart	Day 90	0.277 ± 0.034	0.269 ± 0.023	0.264 ± 0.029	0.262 ± 0.021	0.332 ± 0.017	0.322 ± 0.036	0.317 ± 0.026	0.330 ± 0.025
	Day 120 (recovery)	0.262 ± 0.031	n/a	n/a	0.249 ± 0.026	0.301 ± 0.021	n/a	n/a	0.331 ± 0.041
Thymus	Day 90	0.082 ± 0.021	0.076 ± 0.017	0.080 ± 0.007	0.080 ± 0.014	0.103 ± 0.023	0.094 ± 0.018	0.087 ± 0.017	0.103 ± 0.016
	Day 120 (recovery)	0.078 ± 0.012	n/a	n/a	0.073 ± 0.013	0.120 ± 0.010	n/a	n/a	0.103 ± 0.012*
Brain	Day 90	0.376 ± 0.033	0.387 ± 0.040	0.372 ± 0.044	0.373 ± 0.027	0.699 ± 0.048	0.665 ± 0.054	0.676 ± 0.070	0.673 ± 0.047
	Day 120 (recovery)	0.364 ± 0.024	n/a	n/a	0.358 ± 0.030	0.612 ± 0.063	n/a	n/a	0.699 ± 0.039*
Pituitary	Day 90	0.003 ± 0.00	0.002 ± 0.00	0.002 ± 0.00	0.002 ± 0.00	0.005 ± 0.001	0.005 ± 0.001	0.006 ± 0.001	0.005 ± 0.001
	Day 120 (recovery)	0.002 ± 0.001	n/a	n/a	0.002 ± 0.000	0.005 ± 0.001	n/a	n/a	0.006 ± 0.001
Epididymis	Day 90	0.283 ± 0.052	0.306 ± 0.045	0.297 ± 0.034	0.312 ± 0.044	n/a	n/a	n/a	n/a
	Day 120 (recovery)	0.301 ± 0.057	n/a	n/a	0.314 ± 0.106	n/a	n/a	n/a	n/a
Testes	Day 90	0.680 ± 0.082	0.69 ± 0.082	0.647 ± 0.063	0.684 ± 0.079	n/a	n/a	n/a	n/a
	Day 120 (recovery)	0.622 ± 0.060	n/a	n/a	0.631 ± 0.083	n/a	n/a	n/a	n/a
Prostate	Day 90	0.290 ± 0.071	0.247 ± 0.058	0.259 ± 0.049	0.220 ± 0.036	n/a	n/a	n/a	n/a
	Day 120 (recovery)	0.316 ± 0.065	n/a	n/a	0.274 ± 0.064	n/a	n/a	n/a	n/a
Ovaries	Day 90	n/a	n/a	n/a	n/a	0.069 ± 0.011	0.059 ± 0.010	0.050 ± 0.016	0.055 ± 0.016**
	Day 120 (recovery)	n/a	n/a	n/a	n/a	0.070 ± 0.014	n/a	n/a	0.063 ± 0.012
Uterus	Day 90	n/a	n/a	n/a	n/a	0.258 ± 0.069	0.225 ± 0.044	0.277 ± 0.074	0.278 ± 0.054
	Day 120 (recovery)	n/a	n/a	n/a	n/a	0.376 ± 0.228	n/a	n/a	0.314 ± 0.042

d = day; n/a = not applicable.

* Statistically significant difference from control (*P* < 0.05).

** *P* < 0.01.

a Only the control and high-dose (10 mg/kg bw/d) groups were included in the 30-day recovery period of the study. All groups consisted of 10 animals, with the exception of the recovery groups (5 animals /group).

Macroscopic examination of organs and tissues at the end of the dosing period revealed occasional gross findings that were considered to be either incidental or background conditions occurring sporadically or distributed evenly among all groups, including the control group. These included mechanical damage to the nails in males and females of all groups, lymph node enlargement in females of all groups, alopecia in one low-dose female, and ovary findings in one control female (cystic ovary) and one mid-dose female (hemorrhagic ovary). No gross findings were observed following the recovery period, with the exception of one high-dose male presenting with a hyperemic bladder filled with black flakes and stones, hydronephrosis, and atrophy of the left seminal vesicle. Similar observations were not noted in other animals administered MK-7 examined following either the dosing or recovery period.

**Figure 3:**
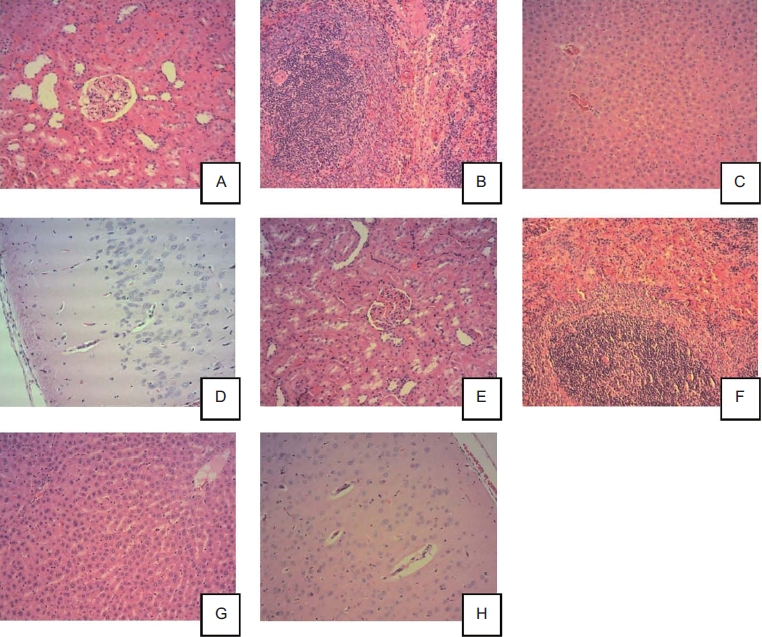
Histopathological pictures of major organs from control animals (A-D) and animals treated with high dose, 10 mg/kg/day menaquinone-7 (MK-7) (E-H). All photos are 10× magnification and all the tissues are considered to be without histopathological findings. Control male rats (10×): renal cortex (A), spleen (B), liver (C), and cerebral cortex (D). MK-7 10 mg/kg/day male rats (10×): renal cortex (E), spleen (F), liver (G), and cerebral cortex (H).

The majority of histological changes noted at the end of the dosing period were incidental or common background findings that were also observed in control animals. These included retinal degeneration, hepatic vacuolation, mild multifocal subacute or chronic inflammation of the liver, heart, lungs, pancreas, urinary bladder, or prostate, mild multifocal subacute vacuolation of the adrenals, mild calculus of the urinary bladder, mild subacute pyelitis of the kidney, and focal thyroid cyst. Figure 3 shows histopathological pictures of major organs from control animals and animals treated with high dose, 10 mg/kg/day MK-7.

## Discussion

The physiological requirements for vitamin K are well-established, and are typically met through dietary intake of vitamin K1 (phylloquinone) from plant sources. Vitamin K2 compounds, known as menaquinones, are less prevalent in the human diet than vitamin K1, but function in the same manner as vitamin K1 (Shearer and Newman, 2008). Menaquinones are also associated with maintaining, in particular, normal bone health (Binkley et al., 2002; Knapen et al., 2007; Bügel, 2008; Shea and Booth, 2008) and, therefore, are promoted for supplemental use. A novel synthetic MK-7 has been developed for human consumption and since no formal toxicological testing on MK-7 has, to our knowledge, been performed, standard toxicological tests were conducted. These included an acute toxicity study and a 90-day subchronic toxicity study in rodents following oral administration of synthetic MK-7.

The results of the acute toxicity study demonstrate that MK-7 is of low acute toxicity in mice following oral exposure, with an LD_50_ of greater than 2000 mg/kg body weight. Subchronic oral exposure of Sprague-Dawley rats to MK-7 also did not produce any mortalities or clinical signs of systemic toxicity at doses of up to 10 mg/kg body weight/day, including any significant effects on body weights, body weight gains, or food consumption.

Compared with control values, significant, yet sporadic, increases in MCV and hematocrit were observed following MK-7 administration, but remained within the normal ranges for Sprague-Dawley rats and were not associated with any other hematological changes related to anemia. These increases were thus considered incidental in nature without toxicological significance. Incidental changes in the clinical chemistry results were similarly observed, with no consistent changes indicative of a specific organ or toxicological effect. Such changes were also neither dose- nor time-dependent, were observed at only one time point and for the most part in one sex only, and were typically within the normal ranges. The few changes that were noted to lie above the normal ranges for Sprague-Dawley rats were observed in female rats at the mid-study point only and were considered to be at the limit of the normal physiological ranges since they were only very slightly increased. Various other hematology and clinical chemistry values, including some control values, were also noted to lie outside of the normal ranges for Sprague-Dawley rats. These were also considered to be at the limit of the normal ranges since they were only very slightly outside of the normal ranges. A compound-related effect could not be attributed to these observations, particularly given the lack of a dose- and/or time-dependent response.

Coagulation time in female rats appeared to be significantly affected by MK-7 administration, with a significant increase in APTT in the mid- and high-dose group compared with the control group at the end of the dosing period. The change in APTT was attributed to the elevated APTT times noted in two female rats in the mid-dose group and one female rat in the high-dose group. Although this observed effect was most likely attributable to suboptimal bleeding technique (as only a few animals were affected), the known role of vitamin K in coagulation was considered. In this regard, vitamin K is well-characterized as being required for blood coagulation, which is inconsistent with an observed increase in clotting time. Furthermore, no significant effects of MK-7 on PT were observed in either females or males, and PT and APTT remained within the normal physiological limits in all groups at the end of the dosing and recovery periods. Thus, the increase in APTT observed was not considered to be compound-related and was further not considered to be adverse in nature. Furthermore, studies in human volunteers demonstrate that natto-derived MK-7 has no effect on the clotting system in healthy individuals. In this regard, single doses of up to 1320 μg of MK-7/person had no effect on blood coagulation or fibrinolytic parameters (thromboelastograph pattern, PT, and APTT) in six healthy men (Sumi, 1999). Likewise, no effects on coagulation (endogenous throm-bin potential and thrombin peak height) were observed in healthy children following a daily intake of 45 μg of natto-derived MK-7 for 8 weeks in a double-blinded, randomized, placebo-controlled study (van Summeren et al., 2009). Large doses of MK-7, however, may interact with anticoagulant therapies that are vitamin K antagonists as demonstrated by Schurgers et al. (2007), and therefore vitamin K2 supplementation should be limited in patients on oral anticoagulants.

At the end of the dosing period, organ weight changes were limited to female rats administered MK-7, and consisted of a decrease in ovary weights in the mid-dose group and an increase in lung weight relative to brain weight in the high-dose group compared with the control group. These significant differences were not dose-dependent and values were typically within the normal ranges. Moreover, no compound-related effects were identified in these or other organs upon macroscopic and histological examination that would be indicative of toxicity. All gross and histological findings were incidental or common background findings that occurred sporadically or were distributed evenly among all groups, including the control group.

Based on the results of the subchronic toxicity study, oral administration of MK-7 does not produce adverse effects in Sprague-Dawley rats at doses of up to 10 mg/kg body weight/day. Thus, the no observed adverse effect level (NOAEL) for synthetic MK-7 in Sprague-Dawley rats is 10 mg/kg body weight/day, the highest dose tested. Considering that supplemental vitamin K is provided at doses in the microgram range, typically 25 to 100 μg/person/day (Physician's Desk Reference, 2008), there is a large margin of safety with synthetic MK-7.

Although no other standard toxicological tests have been identified from the scientific literature that specifically address the safety of MK-7, rat studies investigating the effect of natto-derived MK-7 on bone health have been performed and support the general findings of the present investigation. For instance, dietary administration of MK-7 at a concentration of 18 mg/100 g diet, equivalent to ∼14mg/kg body weight/day, for 24 days had no adverse effects on body weights, femoral dry weight, or other parameters of bone health in ovariectomized female Wistar rats (Yamaguchi et al., 1999). Similar results were observed in female-ovariectomized Wistar rats fed diets containing natto, providing 9.4μg MK-7/100g diet, and supplemented with or without MK-7 for a total of 9.4, 14.1, 18.8, or 37.6μg MK-7/100g diet for periods of 24 to 150 days (Yamaguchi et al., 1999, 2000).

The low oral toxicity of MK-7 is supported by the results of repeated-dose oral toxicity studies conducted on the MK-7 analog MK-4. Such studies include subchronic (13-week) and chronic (1-year) oral toxicity studies conducted in rats and dogs. The NOAEL for MK-4 in dogs was determined to be 200 mg/kg body weight/day in both the 13-week and 1-year studies (Goldsmith et al., 1995; Vanatta et al., 1995). Although no adverse effects were observed in rats orally administered 30 mg MK-4/kg body weight/day for 13 weeks (Doi et al., 1995), the decrease in PT in males rats in the 1-year study (Hosokawa et al., 1995) maybe viewed as a potential adverse effect, with a lowest observed adverse effect level (LOAEL) of 20 mg/kg body weight/day, the lowest dose tested. This value, however, still allows for a large margin of safety for menaqui-nones given the typical supplemental doses of vitamin K and taking into consideration that a NOAEL of 10 mg/kg body weight/day has been determined for synthetic MK-7 in the present investigation. Furthermore, similar effects on blood coagulation have not been observed in healthy humans as noted above.

Although not prevalent in the normal human diet, natural dietary sources of MK-7 include certain cheeses, pork, certain fish, and sauerkraut (Schurgers and Vermeer, 2000). The most abundant dietary source of MK-7, however, is natto, a traditional lapanese food, which contains ∼1 mg MK-7/100g (Schurgers and Vermeer, 2000). Thus, there is a history of human consumption of MK-7, supporting the general safety of this vitamin K2 compound in humans. Moreover, natto-derived MK-7 has been authorized for use in foods for particular nutritional uses, food supplements, and foods intended for the general population in the European Union (EFSA, 2008).

In summary, the results of the present investigation demonstrate that synthetic MK-7 is of low acute oral toxicity, with a median lethal dose (LD_50_) above 2000 mg/kg mice. Furthermore, the NOAEL for MK-7 in Sprague-Dawley rats was considered to be 10 mg/kg body weight/day, the highest dose tested, based on lack of adverse effects observed in the 90-day oral toxicity study. Together with the known history of consumption of natto-derived MK-7 and other menaquinone analogs, these data support the safety of a synthetically derived MK-7 for human consumption at typical supplemental doses of vitamin K.
